# Epidemiology, Species Distribution, Antifungal Susceptibility and Outcome of Nosocomial Candidemia in a Tertiary Care Hospital in Italy

**DOI:** 10.1371/journal.pone.0024198

**Published:** 2011-09-15

**Authors:** Matteo Bassetti, Lucia Taramasso, Elena Nicco, Maria Pia Molinari, Michele Mussap, Claudio Viscoli

**Affiliations:** 1 Infectious Diseases Division, Santa Maria Misericordia University Hospital, Udine, Italy; 2 Infectious Diseases Department, San Martino Hospital and University of Genoa, Genoa, Italy; 3 Bacteriology Unit, San Martino Hospital, Genova, Italy; Los Angeles Biomedical Research Institute, United States of America

## Abstract

*Candida* is an important cause of bloodstream infections (BSI), causing significant mortality and morbidity in health care settings. From January 2008 to December 2010 all consecutive patients who developed candidemia at San Martino University Hospital, Italy were enrolled in the study. A total of 348 episodes of candidaemia were identified during the study period (January 2008–December 2010), with an incidence of 1,73 episodes/1000 admissions. Globally, *albicans* and non-*albicans* species caused around 50% of the cases each. Non-*albicans* included *Candida parapsilosis* (28.4%), *Candida glabrata* (9.5%), *Candida tropicalis* (6.6%), and *Candida krusei* (2.6%). Out of 324 evaluable patients, 141 (43.5%) died within 30 days from the onset of candidemia. *C. parapsilosis* candidemia was associated with the lowest mortality rate (36.2%). In contrast, patients with *C. krusei* BSI had the highest mortality rate (55.5%) in this cohort. Regarding the crude mortality in the different units, patients in Internal Medicine wards had the highest mortality rate (54.1%), followed by patients in ICU and Hemato-Oncology wards (47.6%).

This report shows that candidemia is a significant source of morbidity in Italy, with a substantial burden of disease, mortality, and likely high associated costs. Although our high rates of candidemia may be related to high rates of BSI in general in Italian public hospitals, reasons for these high rates are not clear and warrant further study. Determining factors associated with these high rates may lead to identifying measures that can help to prevent disease.

## Introduction

*Candida* is an important cause of bloodstream infections (BSI), causing significant mortality and morbidity in health care settings. The incidence of candidemia is growing with the increasing complexity of surgical procedures, the existence of patient populations at higher risk of infection, and the changes in patient demographic characteristics. Its overall incidence raised fivefold in the past ten years and *Candida spp.* is currently between the fourth and the sixth most common nosocomial bloodstream isolate in American and European studies [1], [2]. However, candidemia rates vary geographically. For example, an increasing incidence of candidemia in Iceland was reported for the period between 1980 and 1999 [3], but the same was not observed in Switzerland, where a national surveillance study showed that the incidence of candidemia had remained unchanged during the period of 1991 to 2000 [2]. It therefore seems that differences do exist in the epidemiology of candidemia between different countries, underscoring the need for continuous surveillance to monitor trends in incidence, species distribution, and antifungal drug susceptibility profiles. The epidemiology of candidemia has been studied extensively in the United States, Europe, and some countries in South America.

Candidemia remains associated with high crude and attributable mortality rates and with increased costs of care and duration of hospitalization. Attributable mortality has been reported to range from 5% to 71%, and crude mortality rates have been reported to be as high as 81% [4], [5]. In terms of species of *Candida*, recently, a shift towards *non-albicans* species was reported by some authors especially in haematological, transplanted and intensive care unit (ICU) patients [6]–[8].

A reduced antifungal susceptibility in *non-albicans* species and a correlation with routine fluconazole prophylactic use has been suggested [7], [9]. Intrinsic and emerging resistance to azoles actually represents a major challenge for empirical therapeutic and prophylactic strategies [10].

This study was performed to evaluate contemporary epidemiology, species distribution, antifungal susceptibly and outcome of candidema BSI in an Italian hospital.

## Methods

All consecutive patients who developed candidemia at San Martino University Hospital, Italy, a 1,500 beds tertiary care hospital with about 70,000 admissions per year were enrolled in the study during the period January 2008–December 2010. Patients with at least one positive blood culture for Candida spp. And a compatible clinical illness were identified through the microbiological laboratory data base and all informations was recorded in an electronic database. For each patient, only the first episode of candidemia was recorded. Detailed information with regard to candidemia episodes were analyzed, including underlying patient characteristics, the specific fungal pathogen and species, resistance to antifungals and survival. Patients whose cultures grew >1 species of Candida were excluded from the analysis. Patients with candidemia were followed prospectively for 30 days or until their discharge from the hospital. Outcome was recorded only for patients with at least 30 days follow-up after the initial episode of candidemia.

During the study period there were no changes in microbiological laboratory techniques. *Candida* species were isolated from blood using BACTEC 860 system (Becton Dickinson, INC, Sparks, MD). The species were identified using API-32C system (bioMerieux Vitek, Inc, St. Louis, MI). Antifungal susceptibility testing of isolates of Candida spp. was performed by the reference broth microdilution method described by the CLSI [11] . The interpretive breakpoints used for tests were based on values recommended by the CLSI and EUCAST [11], [12].

The following antifungal drugs were tested: amphotericin B, caspofungin, fluconazole, itraconazole and voriconazole.

The Chi-square-test or the Fisher Exact-test were used to compare categorical variables.

The study was approved by the local institutional review board (Comitato Etico, Azienda Ospedaliera Universitaria San Martino) and written patient consent was not required because of the observational nature of this study.

## Results

A total of 348 episodes of candidaemia were identified during the study period (January 2008–December 2010), with an incidence of 1,73 episodes/1000 admissions as shown in table 1. Incidence of candidemia increased from 1,18 in 2008 to 2,37 episodes/1000 admission in 2010 (Table 1). The demographic and clinical characteristics of the patients are summarized in table 1. The majority of patients (93,1%) had one or more comorbidity at the time of the diagnosis of candidaemia. One hundred fifty-seven patients (45,1%) had urdergone a surgical intervention, 143 (41,1%) had solid tumor, 39 (11,2%) were diabetic, 27 (7,7%) had hematologic malignancies, 5 (1,4%) had received a solid organ transplantation and 3 (0,9%) had human immunodeficiency virus infection.

**Table 1:** Patient characteristics and incidence (episode/1000 person/day).

	*Candida* species						
	*C. albicans*	*C.parapsilosis*	*C.glabrata*	*C.tropicalis*	*C. krusei*	Other	All
	(n = 170)	(n = 99)	(n = 33)	(n = 23)	(n = 9)	(n = 14)	(n = 348)
	48,9%	28,4%	9,5%	6,6%	2,6%	4,0%	100,0%
**Patients characteriristic**							
Mean age (standard deviation)	70,1 (±15,72)	66,6 (±16,03)	69,45 (±13,4)	73,35 (±15,32)	47,0 (±15,73)	67,07 (±20,72)	**68,7 (±15,95)**
Male sex, n (%)	99 (57,89)	49 (49,49)	18 (54,54)	13 (56,52)	6 (66,67)	8 (57,14)	**185 (53,31)**
**Underline diseases, n (%)**							
- Surgery	75 (47,77)	54 (34,4)	13 (8,28)	9 (5,73)	2 (1,27)	4 (2,55)	**157**
- Solid organ transplantation	2 (40)	2 (40)	0 (0)	0 (0)	0 (0)	1 (20)	**5**
- Hematologic malignancy	11 (40,75)	7 (25,93)	1 (3,7)	1 (3,7)	6 (22,22)	1 (3,7)	**27**
- HIV	1 (33,33)	0 (0)	0 (0)	1 (33,33)	0 (0)	1 (33,33)	**3**
- Solid tumor	52 (52,53)	30 (30,3)	8 (8,08)	5 (5,05)	0 (0)	4 (4,04)	**99**
- Cardiovascular disease	73 (51)	40 (28)	10 (7)	10 (7)	2 (1,4)	8 (5,6)	**143**
- Diabetes mellitus	20 (51,28)	11 (28,20)	2 (5,13)	4 (10,26)	0 (0)	2 (5,13)	**39**
**Incidence (episodes/1000 admissions)**							
2008	0,55	0,39	0,08	0,08	0,01	0,07	**1,18**
2009	0,95	0,48	0,11	0,1	0,06	0,07	**1,77**
2010	1,09	0,64	0,33	0,17	0,07	0,07	**2,37**

**Figure 1:**
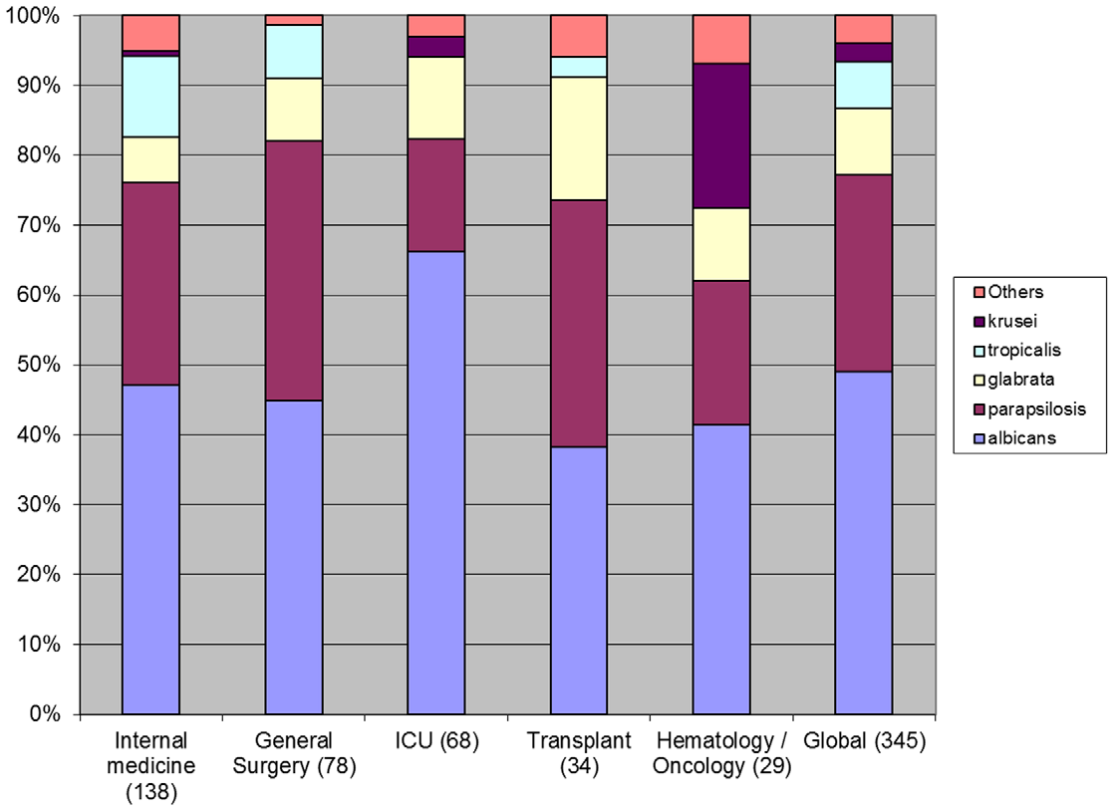
Distribution of the *Candida* species according to underlying pathology/medical care (n).

The distribution of isolated *Candida species* is shown in figure 1. Globally, albicans and non-albicans species caused around 50% of the cases each. Non-albicans included *Candida parapsilosis* (28.4%), *Candida glabrata* (9.5%), *Candida tropicalis* (6.6%), and *Candida krusei* (2.6%). The distribution of albicans and non-albicans strains differed according to the type of patient population and risk factors, as shown in Figure 1. In transplant patients, *C. albicans* was isolated in 40% of the cases, *Candida parapsilosis* in 33% and *Candida glabrata* in 18%; in Hemato-Oncology *C. albicans* accounted for 40% of the cases and *C. parapsilosis* and *C. krusei* for 20% each; on the other hand in ICU *C. albicans* was isolated in 66% of the cases.

Table 2 shows the results of the *in vitro* activity of 5 systemically active antifungal agents tested against 342 BSI isolates of *Candida spp.* Based on CLSI and EUCAST breakpoints [11], [12]. The rate of susceptibility to fluconazole was 98.7% and 96,4% for *C. albicans* and 100% and 98% for *C. parapsilosis*, according to CLSI and EUCAST breakpoints, respectively. Decreased susceptibility to fluconazole was mostly seen with *C. glabrata* [81.8% and 93,9% susceptible in a dose-dependent manner (SDD) or resistant (R) for CLSI and EUCAST, respectively] and *C. tropicalis* (14.3% and 26% SDD or R, for CLSI and EUCAST). Overall, 12.6% with CLSI and 16,4% with EUCAST method of the 342 isolates tested were either SDD or resistant to fluconazole. Caspofungin demonstrated potent activity against *C. albicans*, *C. glabrata* and *C. tropicalis* with higher MICs *C. parapsilosis* (MIC_90_ 1 µg/mL). Resistance to caspofungin was not found with CLSI method. The rates of SDD or R to itraconazole is particularly high in *Candida spp*. (31%).

**Table 2:** Antifungal susceptibility test results for selected species of *Candida* isolated during the study period.

Species (n)	Antifungal agent	MIC range(µg/ml)	MIC_50_(µg/ml)	MIC_90_(µg/ml)	No. (%) of resistant or SDD isolates	
					CLSI	EUCAST
***C. albicans*** ** (167)**	Amfotericin B	0,016–1	0,5	0,5	na	0
	Caspofungin	0,002–0,25	0,06	0,125	0	Na
	Fluconazole	0,125–256	0,25	1	2 (1,3)	6 (3,6)
	Itraconazole	0,006–256	0,125	0,25	18 (11,9)	Na
	Voriconazole	0,002–256	0,003	0,016	2 (1,3)	6 (3,6)
***C. parapsilosis*** ** (98)**	Amfotericin B	0,008–1	0,5	0,5	na	0
	Caspofungin	0,03–1	0,5	1	0	Na
	Fluconazole	0,06–8	1	4	0	2(2)
	Itraconazole	0,006–0,5	0,125	0,25	23 (26,4)	Na
	Voriconazole	0,006–0,125	0,016	0,06	0	0
***C. glabrata*** ** (33)**	Amfotericin B	0,03–1	0,05	1	na	0
	Caspofungin	0,006–0,25	0,06	0,125	0	Na
	Fluconazole	0,5–256	16	32	27 (81,8)	31 (93,9)
	Itraconazole	0,125–16	1	8	29 (96,6)	Na
	Voriconazole	0,006–8	0,25	1	3 (9,6)	16(48,5)
***C. tropicalis*** ** (23)**	Amfotericin B	0,06–2	0,5	1	na	0
	Caspofungin	0,016–0,16	0,03	0,125	0	Na
	Fluconazole	0,25–256	1	8	3 (14,3)	6 (26)
	Itraconazole	0,125–16	0,25	0,5	20 (95,2)	Na
	Voriconazole	0,006–16	0,06	0,5	2 (9,5)	7 (30,4)
**All ** ***Candida spp.*** ** (342)**	Amphotericin B	0.008–1	0.5	0.5	na	0
	Caspofungin	0.002–1	0.06	0.125	0	Na
	Fluconazole	0.06–256	0.5	4	43 (12,6)	56 (16,4)
	Itraconazole	0.006–256	0.125	0.5	106 (31)	Na
	Voriconazole	0.006–256	0.06	0.5	4 (1.2)	34 (9,9)

SDD: susceptible dose dependence; na: breakpoint not available.

CLSI: Clinical and Laboratory Standards Institute; EUCAST: European Committee on Antimicrobial Susceptibility Testing.

Patient outcomes 30 days stratified by Candida species and type of units are reported in table 3. Out of 324 evaluable patients, 141 (43.5%) died within 30 days from the onset of candidemia. *C. parapsilosis* candidemia was associated with the lowest mortality rate (36.2%).In contrast, patients with *C. krusei* BSI had the highest mortality rate (55.5%) in this cohort. No statistically significant differences were observed.

**Table 3:** Number of death and 30 days/mortality for various *Candida spp*. and in the different hospital wards.

	*C. albicans*		*C. glabrata*		*C. krusei*		*C. parapsilosis*		*C. tropicalis*		Others		All	
	Death/N of episodes	Mortality(%)	Death/N of episodes	Mortality(%)	Death/N of episodes	Mortality(%)	Death/N of episodes	Mortality(%)	Death/N of episodes	Mortality(%)	Death/N of episodes	Mortality(%)	Death/N of episodes (%)	MortalityN. (%)
**Hospital Ward**														
Internal Medicine	31/62	(50)	5/8	(62,5)	1	(0)	19/39	(48,7)	8/16	(50)	4/7	(57,1)	**133 (41)**	**68 (51,1)**
Hematology/Oncology	4/8	(50)	0	(0)	6	(50)	2/5	(40)	0	(0)	2/2	(100)	**21 (6.5)**	**10(47,6)**
Transplant	4/12	(33,3)	2/5	(40)	0	(0)	4/12	(33,3)	1	(0)	1/2	(50)	**32 (9.9)**	**11 (34,4)**
Intensive Care Unit	19/42	(45,2)	5/6	(83,3)	2	(100)	4/11	(36,4)	0	(0)	2	(0)	**63 (19.4)**	**30 (47,6)**
General Surgery	12/34	(35,2)	1/7	(14,3)	0	(0)	5/27	(18,5)	3/6	(50)	1/1	(100)	**75 (23)**	**22 (29,3)**
**Global**	**70/158**	**(44,3)**	**13/26**	**(50)**	**5/9**	**(55,5)**	**34/94**	**(36,2)**	**11/23**	**(47,8)**	**8/14**	**(57,1)**	**324 (100)**	**141 (43,5)**

No statistically significant differences were observed with *C. tropicalis*, *C. krusei*, or other *Candida* species.

Regarding the crude mortality in the different units, patients in Internal Medicine wards had the highest mortality rate (54.1%), followed by patients in ICU and Hemato-Oncology wards (47.6%).

## Discussion

Several studies have shown a substantial increase in the incidence of candidemia in the past 2 decades. Our data show that in our hospital the incidence of candidemia has increased steadily and significantly in the past 3 years. Our rates are higher than those reported for centers in the Northern Hemisphere, including the United States (0.28 to 0.96 case per 1,000 admissions) [13], [14], Canada (0.45 case per 1,000 admissions) [15] and some European countries (0.20 to 1.09 case per 1,000 admissions) [16]–[18] and much higher than those reported in Finland (0.026 to 0.03 case per 1,000 admission [19]. The differences in candidemia rates between countries may reflect differences in representativeness and age distributions of the study populations, variations in health care practices, patterns using blood cultures, and antibiotic usage as well as the resistance situation.

Over the past 10 years, some studies have reported a shift in the etiology of candidemia. While *C. albicans* is still considered the most common species causing candidemia, increasing rates of candidemia caused by *C. tropicalis*, *C. parapsilosis*, *C. glabrata*, and *C. krusei* have been reported worldwide [6]–[8], [20]. The reasons for the emergence of non-*C. albicans* species are not completely understood, but some medical conditions may consistently impact the risk of developing candidemia due to non-*C. albicans* species: *C. parapsilosis* fungemia has been associated with vascular catheters and parenteral nutrition [21]. *C. tropicalis* candidemia is associated with cancer and neutropenia [22], and *C. krusei* and *C. glabrata* fungemias are associated with previous exposure to azoles [9], [23]. The findings from our surveillance are partially supportive of these reports. We observed a light predominance of non– *C. albicans Candida* species (51.1%), however *C. albicans* was the most frequently isolated species (48.9%). Our series clearly consolidates the concept that candidemia due to *C. krusei* is rare in Italy and shows that *C. parapsilosis* accounts for the large majority of non-*C. albicans* species. Why *C. krusei* is unusual in Italy is not clear, but the wide geographic variability in the species distribution suggests that factors other than the use of fluconazole may be important, including demographic characteristics and the use of antibiotics. However, although the proportion of *C. krusei* infections in Italy is small, the burden of *this species* is more or less similar to rates reported recently in Spain [18].

We report a low rate of *C. tropicalis* candidemia similar to those published in European and North American series (2 to 10% in Europe and 10 to 12% in the United States and Canada) [24], [25]. Traditionally, *C.tropicalis* has been the second and *C.glabrata* the third or fourth most common Candida species recovered from blood [6], [7].

In our study *C.parapsilosis* surpassed the other non-albicans to become the most common species isolated after *C.albicans*. The high incidence of *C.parapsilosis* candidemia has been previously reported in South American and Italian hospitals [26], [27].

*C. albicans* dominated in our study only in ICU with 66% of the species isolated and is probably related to the restricted fluconazole prophylaxis initiated in 2005 as already published [9]. Non-albicans Candida occurs frequently among haemato-oncologial and in solid organ transplant patients, confirming previous observations [28].

Interesting differences emerged in the profiles of patients with candidal BSI between our data and those previously described. Canadian figures [29] identified that 64% of episodes of nosocomial fungaemia occurred in patients in ICU. In contrast, only 19,4% of episodes in our study arose in ICU patients; this proportion being only a small amount lower than that which arose from patients in the surgical units (23%). In our study in contrast with other experiences, an unusually large proportion (41%) occurred among patients in a general internal medicine service. We hypothesise that this variety of patterns reflects differences in the organisation and resourcing of healthcare delivery in various countries rather than significant differences in the characteristics of the different populations studied.

Antifungal resistance was a rare finding in our study and was restricted to azoles. As with a recently Spanish study [18], none of our Candida bloodstream isolates had MICs of >2 µg/ml for amphotericin B. Our proportion of fluconazole-resistant or SDD isolates (12,6% with CLSI and 16,4% with EUCAST breakpoints) was higher than the rates observed with European (6,3%) and North American (6.6%) isolates [18], [30]. Voriconazole was the azole which exhibited the best in vitro antifungal activity. As reported by others [31] caspofungin demonstrated excellent activity. Important differences in susceptibility, especially for azoles were observed when the CLSI and EUCAST reference methods have been compared [11], [12].

Retrospective cohort studies involving patients with candidemia and varying underlying diseases have revealed worldwide crude and attributable mortality rates of 30%–81% and 5%–71%, respectively [24], [32]–[34]. The severity of candidaemia is confirmed by the high crude mortality rate found in the ECMM survey (38%) [16] as well as in Finland (35%) [35] and in the Barcelona area (44%) [17]. In our series, patients with candidemia had a crude 30 days mortality rate of 43.5%, similar to that reported in Spain. Similar to other reports, patients with C. parapsilosis candidemia had the lowest death rates [25], [36] while *Candida krusei*, *C. glabrata* and *C. tropicalis* BSIs appeared particularly severe, with an unfavourable outcome in more than 40% of patients [5], [16]. This high crude mortality rate of BSIs caused by these species may be due to their occurrence in patients with underlying life-threatening conditions.

Mortality rate at 30 days was higher in Internal Medicine Department (51,1%) and lower in surgical patients (29,3%). These data are different with those reported in other similar studies were the highest mortality has been found in ICU or tumour and haematology patients [37], [38]. Certainly the severity of the underlying medical condition greatly influences the crude mortality rate in these patient populations, however for the patient in Internal Medicine inappropriate therapy could represent an important variable – consisting mostly of omission of initial empirical therapy and an inadequate choice of antifungals – which has been associated with increased mortality [39], [40]. Some limitations of the study must be stressed. The major one is that this is a single centre study and even if it comes from a very large Italian hospital, regional conditions such as features of the patient population and antimicrobial/infection control practices of this specific tertiary care centre may influence the results.

This report shows that candidemia is a significant source of morbidity in Italy, with a substantial burden of disease, mortality, and likely high associated costs. Although our high rates of candidemia may be related to high rates of BSI in general in Italian public hospitals, reasons for these high rates are not clear and warrant further study. Determining factors associated with these high rates may lead to identifying measures that can help to prevent disease.
